# A modal definition of ideal alveolar oxygen

**DOI:** 10.14814/phy2.15787

**Published:** 2023-08-23

**Authors:** Philip J. Peyton

**Affiliations:** ^1^ Department of Critical Care, Anaesthesia, Perioperative and Pain Medicine Program, Melbourne Medical School University of Melbourne Melbourne Victoria Australia; ^2^ Department of Anaesthesia Austin Health Melbourne Victoria Australia; ^3^ Institute for Breathing and Sleep Melbourne Victoria Australia

## Abstract

In the three‐compartment model of lung ventilation‐perfusion heterogeneity (VA/Q scatter), both Bohr dead space and shunt equations require values for central “ideal” compartment O_2_ and CO_2_ partial pressures. However, the ideal alveolar gas equation most accurately calculates mixed (ideal and alveolar dead space) PAO_2_. A novel “modal” definition has been validated for ideal alveolar CO_2_ partial pressure, at the VA/Q ratio in a lung distribution where CO_2_ elimination is maximal. A multicompartment computer model of physiological, lognormal distributions of VA and Q was used to identify modal “ideal” PAO_2_, and find a modification of the alveolar gas equation to estimate it across a wide range of severity of VA/Q heterogeneity and FIO_2_. This was then validated in vivo using data from a study of 36 anesthetized, ventilated patients with FIO_2_ 0.35–80. Substitution in the alveolar gas equation of respiratory exchange ratio R with $\;\text{modalR}=R–\left(1–\mathrm{PEtC}O_{2}/P{\mathrm{aCO}}_{2}\right)$ achieved excellent agreement (*r* ^2^ = 0.999) between the calculated ideal PAO_2_ and the alveolar‐capillary Pc'O_2_ at the modal VO_2_ point (“modal” Pc'O_2_), across a range of log standard deviation of VA 0.25–1.75, true shunt 0%–20%, overall VA/Q 0.4–1.6, and FIO_2_ 0.18–1.0, where the modeled PaO_2_ was over 50 mm Hg. Modal ideal PAO_2_ can be reliably estimated using routine blood gas measurements.

## 1 INTRODUCTION

Heterogeneity of alveolar ventilation ($\dot{V}A$) and blood flow ($\dot{Q}$) ratios across the lung ($\dot{V}A/\dot{Q}$ scatter) is commonly described using the three‐compartment or “Riley” model, described over 75 years ago. This simple model depicts all alveolar‐capillary lung gas exchange taking place within a theoretical, central, “ideal” lung compartment which has uniform blood flow and ventilation. The ideal compartment sits between an unperfused alveolar dead space compartment and a third, unventilated, venous admixture, or shunt compartment. (Riley et al., 1946) This framework provides two simple and familiar mixing equations that allow $\dot{V}A/\dot{Q}$ scatter to be quantified, the Bohr dead space equation, and the shunt equation of Berggren (Berggren, 1942; Bohr, 1891).

Solution of the Bohr equation and the shunt equation requires a value for the alveolar partial pressure of carbon dioxide (CO_2_) or oxygen (O_2_), respectively, in the ideal compartment. However, there have always been difficulties in defining ideal alveolar gas partial pressure within this model. The Enghoff modification of the Bohr equation substitutes this unknown quantity with arterial CO_2_ partial pressure ($P{\mathrm{aCO}}_{2}$) as a readily measurable approximation for ideal alveolar CO_2_ partial pressure, but $P{\mathrm{aCO}}_{2}$ in fact represents the combined CO_2_ content of the theoretical ideal and shunt compartments (Enghoff, 1938).

Estimation of ideal alveolar O_2_ partial pressure ($P{\mathrm{AO}}_{2}\text{ideal}$) and blood O_2_ content in the ideal compartment customarily uses the alveolar gas equation, (Nunn, 1993; Riley & Cournand, 1949)

(1) $$\begin{array}{c} P{\mathrm{AO}}_{2}\text{ideal}=\mathrm{FI}O_{2}∙\mathrm{PB}-P{\mathrm{ACO}}_{2}i\text{deal}/R\\ \;-\left[\mathrm{FI}O_{2}∙P{\mathrm{ACO}}_{2}\text{ideal}∙\left(1-1/R\right)\right] \end{array}$$

where $P{\mathrm{ACO}}_{2}$ *ideal* is the alveolar CO_2_ partial pressure in the ideal compartment, PB barometric pressure, $\mathrm{FI}O_{2}$ the fractional inspired O_2_ concentration, and R the respiratory exchange ratio (the term on the right in square brackets is frequently omitted for simplicity). This convention has been followed in many previous studies measuring shunt fraction (Nunn, 1993; Peyton et al., 2004, 2005; West et al., 2020). Indeed, this equation is still frequently referred to as the “ideal alveolar gas equation” (West et al., 2020). For practicality of measurement, $P{\mathrm{aCO}}_{2}$ is often used instead of $P{\mathrm{ACO}}_{2}$

(1a) $$\begin{array}{c} P{\mathrm{AO}}_{2}\text{ideal}=\mathrm{FI}O_{2}∙\mathrm{PB}-P{\mathrm{aCO}}_{2}/R\\ \;-\left[\mathrm{FI}O_{2}∙P{\mathrm{aCO}}_{2}∙\left(1-1/R\right)\right] \end{array}$$

Since the development by Riley and colleagues of the three‐compartment model, (Riley et al., 1946; Riley & Cournand, 1949) advances in technology and computing have allowed us to model and study physiologically realistic, “lognormal” patterns of distribution of $\dot{V}A$ and Q, and of gas exchange for O_2_ and CO_2_ and other gases, across the range of $\dot{V}A/\dot{Q\;}$ ratios throughout the lung. These models provide much better and more sophisticated understanding of gas exchange for gases of different solubilities, but challenge some existing assumptions of three‐compartment theory. For example, it has been shown in patients under inhalational anesthesia how gases with differing solubilities have widely different alveolar dead space volumes, and therefore different effective or “ideal” alveolar ventilation rates, simultaneously within the same $\dot{V}A/\dot{Q}$ distribution (Peyton et al., 2020).

Derivation of the alveolar gas equation (see Appendix 1C), based on mass balance principles, assumes that all O_2_ uptake ($\dot{V}O_{2}$) and CO_2_ elimination ($\dot{V}{\mathrm{CO}}_{2}$) takes place within the same “ideal” compartment. However, due to their widely differing solubilities in blood, $\dot{V}O_{2}$ and $\dot{V}{\mathrm{CO}}_{2}$ distributions are not colocated across the range of $\dot{V}A/\dot{Q}$ ratios in the lung, and their divergence increases as $\dot{V}A/\dot{Q}$ scatter worsens (Farhi, 1967; Farhi & Yokoyama, 1967; Peyton et al., 2020). O_2_ uptake takes place predominantly in lower $\dot{V}A/\dot{Q}$ ratio lung units, whereas elimination of CO_2_, which is more highly soluble, takes place largely in well ventilated lung regions. This undermines the fundamental assumption on which the concept of a common central “ideal” compartment containing all gas exchange is based.

Recently, an alternative definition of ideal alveolar gas has been proposed based upon physiological, lognormal distributions of $\dot{V}A$ and Q, instead of the three‐compartment model (Peyton, 2021). Ideal alveolar gas partial pressure for any gas species is defined as that found at the $\dot{V}A/\dot{Q}$ ratio where the gas exchange rate for that gas is maximal or “modal” across the lung. This $\dot{V}A/\dot{Q}$ point is determined by the solubility of the gas in blood, and is widely different for different gases.

For CO_2_, with a relatively linear dissociation curve, the alveolar‐capillary partial pressure at the $\dot{V}A/\dot{Q}$ ratio of modal CO_2_ elimination (modal *ideal* $P{\mathrm{ACO}}_{2}$) has been shown to equal the mean of the measured arterial and end‐tidal CO_2_ partial pressures (Peyton, 2021). This is also the case for any inert gas. Due to its alinear dissociation curve, however, this relationship does not hold for O_2_.

In the current study, data from a physiological, multicompartment computer model of lung $\dot{V}A/\dot{Q}$ scatter was used to seek a modification of the alveolar gas equation that would provide a practical estimate of modal *ideal* alveolar O_2_ partial pressure (modal *ideal* $P{\mathrm{AO}}_{2}$), at the $\dot{V}A/\dot{Q}$ ratio of maximal (modal) O_2_ uptake rate. The mass balance principle described by Equation 1 applies to any $\dot{V}A/\dot{Q}$ compartment in the lung (see Appendix 1B) if R within that compartment is known. Therefore, this required characterization, within any given distribution of lung $\dot{V}A$ and $\dot{Q}$, of the particular value of R in Equation 1 at the $\dot{V}A/\dot{Q}$ ratio where the modal *ideal* $P{\mathrm{AO}}_{2}$ is located (modal R). The resulting equation should be generalizable across a wide range of severity of $\dot{V}A/\dot{Q}$ heterogeneity and of $\mathrm{FI}O_{2}$. Its ability to accurately predict the modal *ideal* $P{\mathrm{AO}}_{2}$ was therefore tested across a diverse range of modeled scenarios. The concept was illustrated and subsequently validated with modeling of lung gas exchange in a series of anesthetized, ventilated surgical patients, using in vivo clinical data collected in these patients as input and target output modeling variables.

## 2 METHODS

### 2.1 Clinical in vivo data collection

Data were used that was collected following Ethics review and approval and informed patient consent (HREC H99/00798 and HREC/16/Austin/419) at the Austin Hospital, Melbourne, in two cohorts of patients with no history of acute or chronic respiratory disease undergoing cardiac surgery during near steady‐state conditions in the precardiopulmonary bypass period. The earlier cohort recruited six patients and the later cohort 30 patients. Of these, a total of 30 patients were male. The mean (SD) patient age was 66 (10) years and body mass index was 30.5 (5.3). Delivered concentrations of oxygen were set to achieve an $\mathrm{FI}O_{2}$ of 0.5–0.6 in the later cohort, and 0.3–0.5 in the earlier cohort in whom a second set of measurements was performed in the postcardiopulmonary bypass period. These were treated as independent measurements for the purposes of statistical analysis, resulting in a total of 42 measurements. The anesthesia management and data collection protocol is detailed in Appendix 3.

### 2.2 Lung computer modeling

Data collection from multicompartment lung modeling involved two parallel modelling exercises:

A. Systematic modeling was done, using arbitrary input data, of distributions in the lung of $\dot{V}A$, $\dot{Q}$, $\dot{V}A/\dot{Q}$ scatter and the resulting distributions of partial pressures and gas exchange in each lung compartment *n* ($\dot{V}{\mathrm{CO}}_{2}n$, $\dot{V}O_{2}n$ and respiratory exchange ratio R*n*). Values were obtained for variables in the alveolar gas equation within these distributions at the $\dot{V}A/\dot{Q}$ ratio of modal $\dot{V}O_{2}$, and their relationship across a range of theoretical scenarios was characterized.
B. Clinical simulation used the data measurements from patients as inputs and target outputs for the model, to both illustrate the distributions being studied and validate the resulting equation in vivo.

The model of lung gas exchange used physiological distributions of $\dot{V}A$ and $\dot{Q}$ across the lung. These distributions were idealized, unimodal, lognormal distributions across 100 lung compartments *n*, ($\dot{V}An$ and $\dot{Q}n$) with the degree of $\dot{V}A/\dot{Q}$ scatter scalable by the log standard deviation (log SD) of $\dot{V}A$ to represent a wide range of severity of $\dot{V}A/\dot{Q}$ heterogeneity. In addition, the model allows a proportion of the total pulmonary blood flow to be allocated to an additional “true shunt” compartment where no alveolar ventilation and gas exchange occurs. The structure of the model is summarized in Appendix 2 and has been previously described (Kelman, 1966, 1967; Olszowska & Wagner, 1980; Peyton et al., 2001; Siggaard‐Andersen, 1974; West, 1969). Within each lung compartment, alveolar and end‐capillary partial pressure were considered fully equilibrated (alveolar‐capillary partial pressure).

A. Systematic modeling:

Theoretical scenarios were modeled using arbitrary input values, where overall R was 0.8. For each of these theoretical scenarios, the log SD of $\dot{V}A$ was varied in seven increments of 0.25 up to 1.75. The modeling was repeated for scenarios with an overall $\dot{V}A/\dot{Q}$ ratio of 0.4, 0.8, and 1.6, and for R = 1.0. Two further scenarios were modeled where 10% and 20% of total blood flow $\dot{Q}t$ was allocated to true shunt, giving a total of 42 different scenarios. For each of these, six different $\mathrm{FI}O_{2}$ values were modeled, ranging from 0.18 to 1.0.

The outputs calculated by the model were the combined flow‐weighted means of the equilibration partial pressures and end‐capillary blood gas contents, within all alveolar‐capillary lung compartments *n* (including true shunt), to obtain mixed alveolar gas partial pressure (${\mathrm{PAO}}_{2}$ and ${\mathrm{PACO}}_{2}$) and arterial partial pressure ($P{\mathrm{aO}}_{2}$ and $P{\mathrm{aCO}}_{2}$) and blood content ($C{\mathrm{aO}}_{2}$ and $C{\mathrm{aCO}}_{2}$) of O_2_ and CO_2_, as well as the distributions across all lung compartments of alveolar‐capillary equilibration partial pressures and gas exchange of O_2_ (Pc'O2n and $\dot{V}O_{2}n$) and CO_2_ (Pc'CO2n and $\dot{V}{\mathrm{CO}}_{2}n$). From the distributions of $\dot{V}{\mathrm{CO}}_{2}n$ and $\dot{V}O_{2}n$ the respiratory exchange ratio in each lung compartment (Rn) was calculated.

$$Rn=\dot{V}{\mathrm{CO}}_{2}n/\dot{V}O_{2}n.$$

Within each compartment *n*, the same mass balance principle holds as that expressed by the alveolar gas equation for the lung overall (see Appendix 1B)

(1*n*) $$\begin{array}{c} \mathrm{Pc}\;'O_{2}n=\mathrm{FI}O_{2}∙\mathrm{PB}-\mathrm{Pc}\;'{\mathrm{CO}}_{2}n/Rn-\left[\mathrm{FI}O_{2}∙\mathrm{Pc}\;'{\mathrm{CO}}_{2}n∙\left(1-1/Rn\right)\right] \end{array}$$

i) Modal Rn

For each theoretical scenario modeled, the lung compartment of maximal or modal $\dot{V}O_{2}n$ within the distributions of $\dot{V}O_{2}n$ was identified using a peak detector subroutine. The Pc'O2n (modal Pc'O2n) and Rn (modal Rn) in this compartment were recorded. The relationship of modal Rn to overall R in each scenario was examined, and data from all scenarios were combined and plotted. An empirical equation was sought for this relationship (see Results: Equation 3, below), which uses overall R and clinically available gas partial pressure measurements, to provide an estimate of the modal Rn (predicted modal Rn). The agreement of the predicted modal Rn from this equation with the modal Rn identified within each distribution was assessed across all scenarios modeled.

ii) Modal *ideal* $P{\mathrm{AO}}_{2}$

In each scenario, the modal *ideal* $P{\mathrm{AO}}_{2}$ was defined as the Pc'O2n at the modal $\dot{V}O_{2}n$ point within the distributions of $\dot{V}O_{2}n$. This value is therefore predicted by the following modification of Equation 1*n*, using modal Rn (see Equation 3, below) and using $\mathrm{Pa}{\mathrm{CO}}_{2}$ as a substitute for Pc'CO2n:

(2) $$\text{modal}\;\text{ideal}\;P{\mathrm{AO}}_{2}=\mathrm{FI}O_{2}∙\mathrm{PB}-P{\mathrm{aCO}}_{2}/\text{modal}\;Rn-\left[\mathrm{FI}O_{2}∙P{\mathrm{aCO}}_{2}∙\left(1-1/\text{modal}\;Rn\right)\right]$$

The agreement of the modal Pc'O2n identified by peak detection within the distributions with the predicted modal *ideal* $P{\mathrm{AO}}_{2}$ from Equations 2 and 3 (see Results: Equation 2a, below) was assessed across all scenarios combined. The acceptability of use of $\mathrm{Pa}{\mathrm{CO}}_{2}$ as a substitute for Pc'CO2n in this equation was also assessed. In addition, the threshold of arterial hypoxemia (lowest calculated $\mathrm{Pa}O_{2}$) simulated in these scenarios, beyond which agreement deteriorated, was examined.

B. Clinical in vivo modeling:

i) Mean in vivo simulation: To illustrate the nature of the relationship of gas exchange distributions for O_2_ and CO_2_ to distributions of $\dot{V}A/\dot{Q}$ scatter, the mean measured values in the patient sample studied were used as input variables for a multicompartment lung model scenario. The log SD of $\dot{V}A$ and true shunt fraction that most closely approximated the mean measured output variables ($P{\mathrm{AO}}_{2}$, $P{\mathrm{ACO}}_{2}$, $\mathrm{Pa}O_{2}$, and $\mathrm{Pa}{\mathrm{CO}}_{2}$) were determined. The resulting distributions across 100 lung compartments *n* of $\dot{V}An,$ $\dot{Qn}$, gas exchange ($\dot{V}O_{2}n$ and $\dot{V}{\mathrm{CO}}_{2}n$), and alveolar‐capillary partial pressures (Pc'O2n and Pc'CO2n) across the lung were plotted. The modal *ideal* $P{\mathrm{AO}}_{2}$ and modal *ideal* $P{\mathrm{ACO}}_{2}$ were identified.
ii) Validation with individual patient modeling: Using the measured $\mathrm{FI}O_{2}$ as an input variable in each patient, the modal Pc'O2n for each patient was identified by peak detection using the multicompartment lung model. This was then compared with the predicted modal *ideal* $P{\mathrm{AO}}_{2}$ for each patient calculated from combination of Equations 2 and 3 (see Results: Equation 2a below).

### 2.3 Statistical analysis

For the purposes of in vivo validation, the primary statistical comparison was between the predicted modal *ideal* $P{\mathrm{AO}}_{2}$ and the modal Pc'O2n identified by the multicompartment lung model for each patient. Secondary comparisons were made between the $P{\mathrm{AO}}_{2}$ from Equation 1 and the measured end‐tidal O_2_ partial pressure (PE'O2) in the patients. Measured end‐tidal gas partial pressure (PE'O2 and PE'CO2) was considered physiologically equivalent to mixed alveolar gas partial pressure in this population with no history of lung pathology and with no longtitudinal gas flow stratification, manifested as a flat end‐expiratory Phase 3 gas concentration plateau (see Supplementary material for an illustrative example). It was hypothesized that there would be no clinically significant difference between these two variables, consistent with the derivation of the alveolar gas equation (Appendix 1C). Comparisons were also made with the mean alveolar O_2_ partial pressure from Equation 1a, and with the predicted modal *ideal* $P{\mathrm{AO}}_{2}$ from Equation 2.

Comparison of mean values was done with the *t*‐test for paired data for normally distributed or log transformed non‐normal data confirmed with the Shapiro–Wilk test. The Pearson correlation coefficient *r* was used to measure agreement across the range of $\dot{V}A/\dot{Q}$ relationships and $\mathrm{FI}O_{2}$ modeled, as well as the median (and 95th centile) relative error between modeled modal values and calculated values for modal Rn and modal *ideal* $P{\mathrm{AO}}_{2}$. Agreement was also calculated using Bland–Altman analysis for clinical data. All statistical tests were two‐tailed, with a threshold of significance of *p* < 0.05, and analysis was done using Stata 12 (StataCorp, College Station, TX, USA).

## 3 RESULTS

### 3.1 Multicompartment lung computer modeling

B.(i) Mean in vivo simulation:

This modeling scenario is presented first to illustrate the relationships being described in the study. The required modeling input variable are listed in Table 1. The measured mean patient data for these input variables are shown. $\mathrm{FI}O_{2}$ was 0.53 with balance gas nitrogen, hemoglobin concentration was 12.6 g/L, body temperature 35.4°C, with $\dot{V}O_{2}$ of 190 mL/min with overall R of 0.94. Total $\dot{Q}$ ($\dot{Q}t$) was set to 4.6 L/min and $\dot{V}A$ 4.2 L/min to match the measured values in the patient sample. Input values for the model were nominated which were equal to the mean values in Table 1. A simulation with a log SD of $\dot{V}A$ of 1.17 and true shunt fraction of 8.5% was found by an iterative trial and error process to produce outputs from the model ($P{\mathrm{aO}}_{2}$, $P{\mathrm{AO}}_{2}$, $P{\mathrm{aCO}}_{2}$, and $P{\mathrm{ACO}}_{2}$) that most closely approximated the measured mean values in the patients.

**TABLE 1:** Measured variables in patients in the study (n = 41). Data are mean values and standard deviation (SD).

Model inputs		Model outputs	
$\mathrm{FI}O_{2}$	0.53 (0.11)	$P{\mathrm{aO}}_{2}$ mm Hg	192.5 (67.2)
$\dot{Q}t$ l/min	4.6 (1.3)	$PE'O_{2}$ mm Hg	341.1 (79.6)
$\dot{V}A$ l/min	4.2 (1.1)	$P{aCO}_{2}$ mm Hg	40.3 (4.8)
$\dot{V}O_{2}$ l/min	0.190 (0.061)	$PE'{\mathrm{CO}}_{2}$ mm Hg	30.5 (4.4)
$\dot{V}{\mathrm{CO}}_{2}$ l/min	0.178 (0.048)	$S{\mathrm{aO}}_{2}$ %	98.8 (0.6)
Arterial BE mmol/l	0.9 (1.5)	$C{\mathrm{aO}}_{2}$ ml/100 mL	17.5 (2.7)
Arterial pH	7.41 (0.04)	$C\bar{v}O_{2}$ ml/100 mL	13.4 (2.8)
Temperature ^o^C	35.4 (0.7)	VDA/VA %	24.4 (7.6)
Hemoglobin g/dl	12.6 (2.0)	$\dot{Q}s/\dot{Q}t$ %	12.4 (6.8)

*Note*: Variables used as inputs to the lung modeling are listed above, and those that were outputs listed below.

Abbreviations: BE, base excess; $C{\mathrm{aO}}_{2}$, arterial blood O_2_ content; $C\bar{v}O_{2}$, mixed venous blood O_2_ content; Pa, arterial partial pressure; PEʹ, end‐tidal partial pressure; $\dot{Q}t$, total pulmonary blood flow; $\dot{Q}s/\dot{Q}t$, shunt fraction; $S{\mathrm{aO}}_{2}$, arterial hemoglobin O_2_ saturation; $\dot{V}A$, alveolar ventilation rate; $\dot{V}{\mathrm{CO}}_{2}$, CO_2_ elimination rate; VDA/VA, alveolar deadspace fraction; $\dot{V}O_{2}$, O_2_ uptake rate.

**FIGURE 1:**
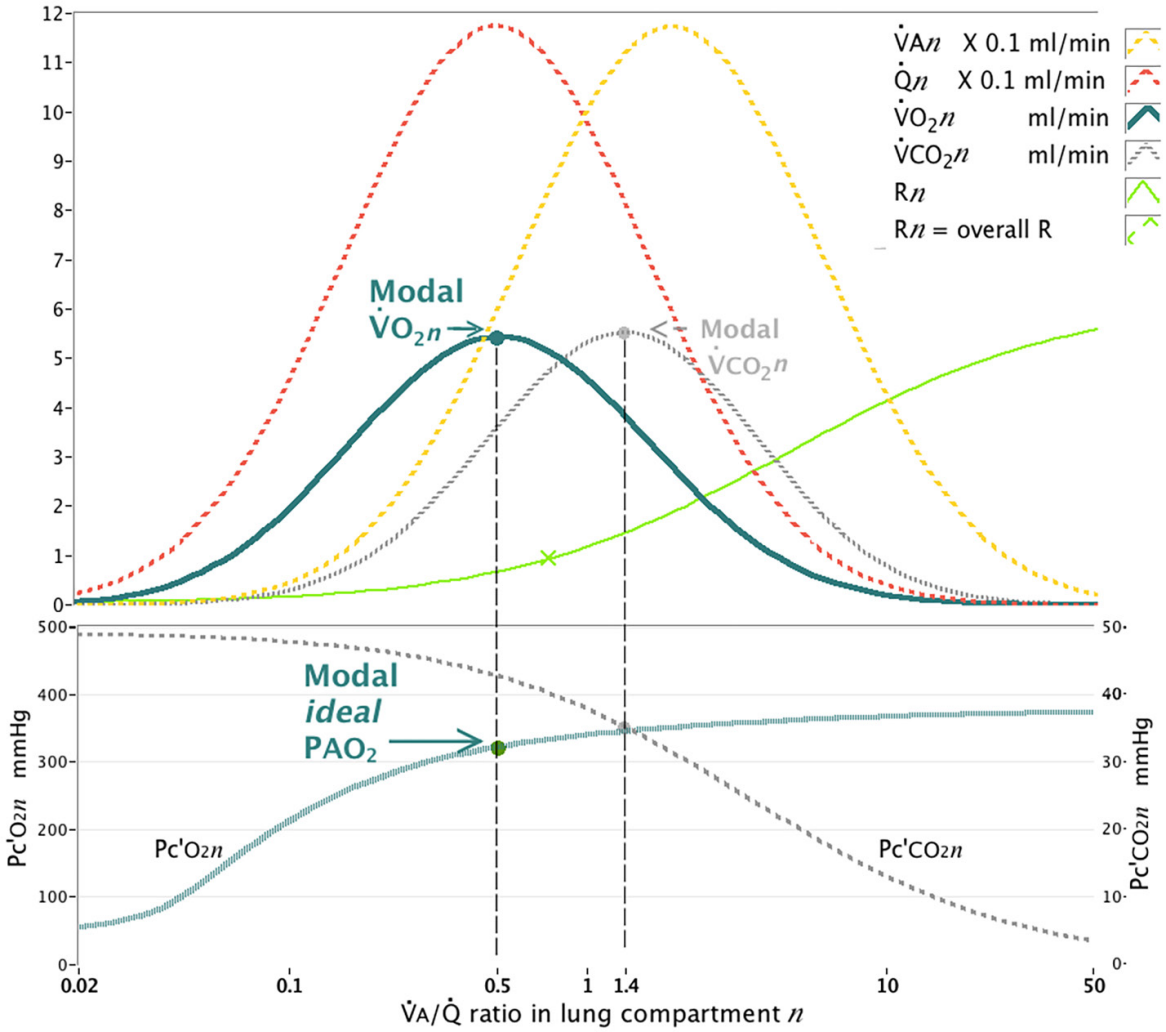
Clinical in vivo mean modeling scenario: Distributions of alveolar ventilation $\dot{V}An$, pulmonary blood flow $\dot{Q}n$ and oxygen uptake $\dot{V}O_{2}n$ and carbon dioxide elimination $\dot{V}{\mathrm{CO}}_{2}n$ across the range of lung $\dot{V}A/\dot{Q}$ ratios generated by the model, to simulate the mean clinical scenario. Hundred lung compartments *n* were modeled. Input data were the means measured from the patients of variables listed in Table 1. Total $\dot{Q}$ ($\dot{Q}t$) was 4.6 L/min and $\dot{V}A$ 4.2 L/min. The log SD of $\dot{V}A$ was 1.17 with true shunt fraction of 8.5%, which produced outputs from the model ($P{\mathrm{aO}}_{2}$, $P{\mathrm{AO}}_{2}$, $P{\mathrm{aCO}}_{2}$, and $P{\mathrm{ACO}}_{2}$) that most closely approximated the measured mean values in Table 1. Also plotted are the corresponding distributions of alveolar‐capillary partial pressures (Pc'O2n and Pc'CO2n) and the respiratory exchange ratio (R*n*) in each lung compartment. The position of the Pc'O2n (321.4 mm Hg) at the modal $\dot{V}O_{2}n$ in this scenario is indicated (modal *ideal* $P{\mathrm{AO}}_{2}$), as well as the modal *ideal* $P{\mathrm{ACO}}_{2}$. The position of the arterial, mixed venous and end‐tidal PCO2 values are shown, as is the $\dot{V}A/\dot{Q}$ ratio where R*n* = overall lung respiratory exchange ratio R of 0.94.

The resulting lung distributions of $\dot{V}An$, $\dot{Q}n$, $\dot{V}{\mathrm{CO}}_{2}n$, and $\dot{V}O_{2}n$ across the range of $\dot{V}A/\dot{Q}$ ratios for this scenario are shown in Figure 1. Also plotted are the corresponding alveolar‐capillary partial pressures (Pc'O2n and Pc'CO2n) and the compartmental respiratory exchange ratio (Rn). The modal $\dot{V}O_{2}n$ point and modal $\dot{V}{\mathrm{CO}}_{2}n$ point in this simulation are indicated.

The position of the Pc'O2n at the modal $\dot{V}O_{2}n$ point (modal *ideal* $P{\mathrm{AO}}_{2}$) was 321.4 mm Hg in this simulation, and is indicated in Figure 1, along with the position of the modal *ideal* $P{\mathrm{ACO}}_{2}$. The Pc'CO2n at the $\dot{V}A/\dot{Q}$ ratio of the modal Pc'O2n point (43.5 mm Hg) more closely approximated the $P{\mathrm{aCO}}_{2}$ (40.3 mm Hg) than did the PE'CO2 (30.5 mm Hg), and the $P{\mathrm{aCO}}_{2}$ was subsequently used in seeking a modification of the alveolar gas equation to calculate the modal *ideal* $P{\mathrm{AO}}_{2}$.

### 3.2 Theoretical scenarios

A.(i) Characterization of modal Rn:

Across the range of scenarios modeled, the Rn value at the modal $\dot{V}O_{2}n$ point (modal Rn) was empirically found to approximate the following relationship to the overall R, which includes a term which reflects the effect of increasing $\dot{V}A/\dot{Q}$ scatter, using the calculated alveolar‐arterial CO_2_ partial pressure gradient for each scenario,

(3) $$\text{modal}\;Rn=R-\left(1-P{\mathrm{ACO}}_{2}/P{\mathrm{aCO}}_{2}\right)$$

The accuracy of Equation 3 to predict the modal Rn deteriorated progressively where the $P{\mathrm{aO}}_{2}$ calculated in the scenario was below 50 mm Hg. Rn becomes relatively fixed in the low $\dot{V}An/\dot{Qn}$ range, as is evident in Figure 1, which is due to the alinearity of the O_2_ dissociation curve relative to that for CO_2_. This was seen in several scenarios where an overall $\dot{V}A/\dot{Q}$ ratio of 0.4 was modeled, and other scenarios at lower $\mathrm{FI}O_{2}$ where a severe degree of $\dot{V}A/\dot{Q}$ scatter (log SD of $\dot{V}A$ of 1.5 or more) or large true shunt fraction (20%) were modeled. Scenarios where the $P{\mathrm{aO}}_{2}$ calculated was below 50 mm Hg was therefore subsequently excluded from the analysis.

**FIGURE 2:**
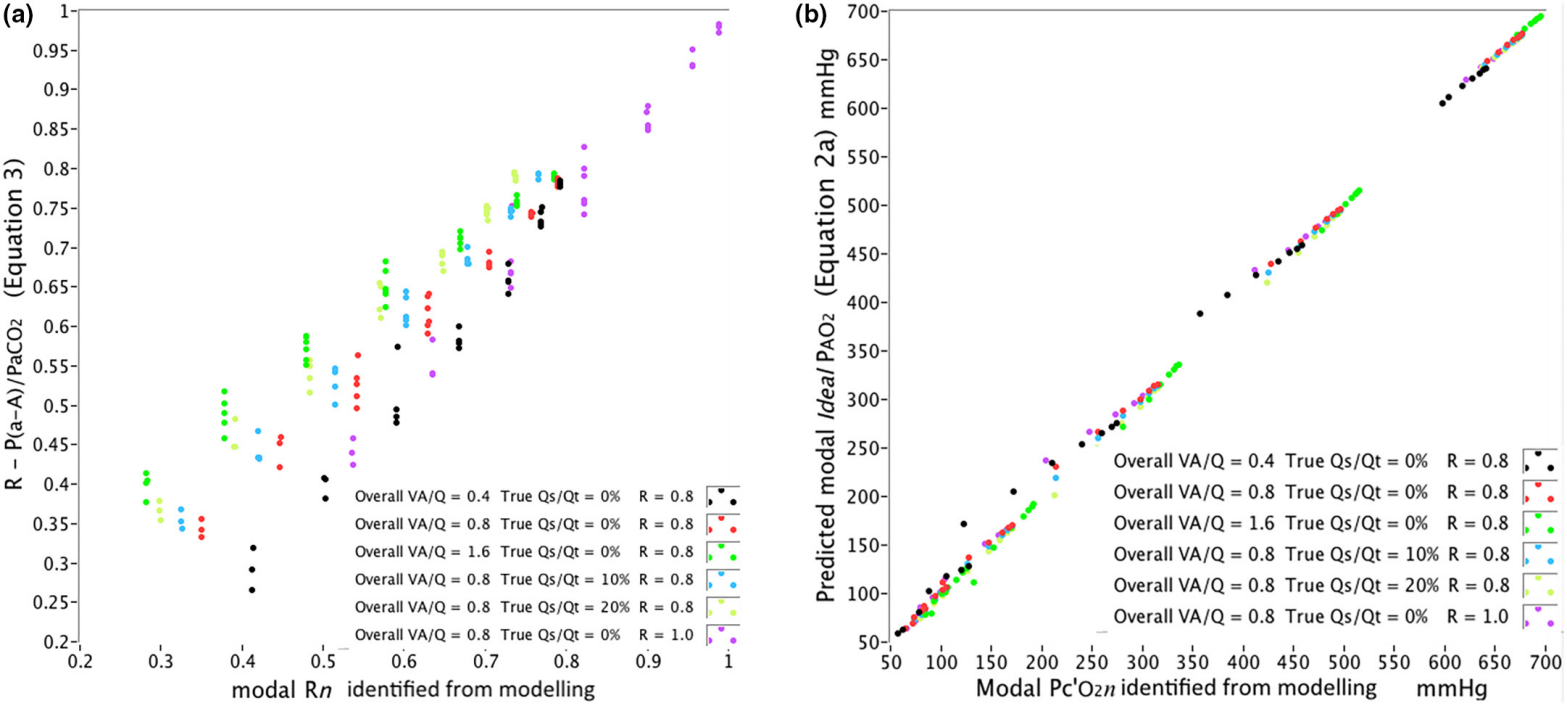
(a) Systematic modeling scenarios: The relationship of the respiratory exchange ratio (R*n*) identified by modeling in the lung compartment at the modal $\dot{V}O_{2}n$ (modal R*n*) to R*n* predicted by Equation 3 across the range of $\mathrm{FI}O_{2}$ modeled from 0.18 to 1.0. At each $\mathrm{FI}O_{2}$, the log standard deviation (log SD) of $\dot{V}A$ was varied in seven increments of 0.25 up to 1.75, repeated for scenarios with an overall $\dot{V}A/\dot{Q}$ ratio of 0.4, 0.8 and 1.6, R = 0.8 and 1.0, and true shunt fraction of 0%, 10% and 20%. Scenarios resulting in $P{\mathrm{aO}}_{2}$ of less than 50 mmHg were excluded. (b) Systematic modeling scenarios: The relationship of the Pc'O2n identified by modeling in the lung compartment at the modal $\dot{V}O_{2}n$ (modal Pc'O2) to modal *ideal* $P{\mathrm{AO}}_{2}$ predicted by Equation a across the range of $\mathrm{FI}O_{2}$ modeled from 0.18 to 1.0. At each $\mathrm{FI}O_{2}$, the log standard deviation (log SD) of $\dot{V}A$ was varied in seven increments of 0.25 up to 1.75, repeated for scenarios with an overall $\dot{V}A/\dot{Q}$ ratio of 0.4, 0.8 and 1.6, R = 0.8 and 1.0, and true shunt fraction of 0%, 10% and 20%. Scenarios resulting in $P{\mathrm{aO}}_{2}$ of less than 50 mm Hg were excluded.

The accuracy of Equation 3 is illustrated in Figure 2 across the range of theoretical scenarios modeled. The predicted modal Rn from Equation 3 is plotted against the modal Rn identified from the distributions in each scenario. The error is summarized in Table 2 at each $\mathrm{FI}O_{2}$ modeled.

**TABLE 2:** Systematic modeling: Agreement (variance *r* ^2^, relative median error (%) and 95th centile) between the respiratory exchange ratio Rn at the $\dot{V}A/\dot{Q}$ ratio of modal $\dot{V}O_{2}n$ identified from distributions of $\dot{V}O_{2}$ calculated by the multicompartment lung model (modal Rn), and the predicted modal Rn from Equation 3, across theoretical scenarios modeled where the resulting $P{\mathrm{aO}}_{2}$ was 50 mm Hg or greater.

$\mathrm{FI}O_{2}$	*r* ^2^	Median error %	95th centile error %
0.18	0.956	2.4	14.9
0.21	0.940	3.3	18.8
0.30	0.904	5.0	20.0
0.50	0.883	5.8	26.9
0.75	0.874	6.7	36.0
1.0	0.889	6.4	27.8

*Note*: $\mathrm{FI}O_{2}$ was varied from 0.18 to 1.0. At each $\mathrm{FI}O_{2}$, the log standard deviation (log SD) of $\dot{V}A$ was varied in seven increments of 0.25 up to 1.75, repeated for scenarios with an overall $\dot{V}A/\dot{Q}$ ratio of 0.4, 0.8 and 1.6, R = 0.8 and 1.0, and true shunt fraction of 0%, 10% and 20%. See Figure 2a.

A.(ii) Modal *ideal* $P{\mathrm{AO}}_{2}$:

Substitution of R in Equation 2 with modal Rn from Equation 3 gives a modified form of the alveolar gas equation that predicts the modal *ideal* $P{\mathrm{AO}}_{2}$.

(2a) $$\begin{array}{c} \;\text{modal}\;\text{ideal}\;P{\mathrm{AO}}_{2}=\mathrm{FI}O_{2}∙\left(\mathrm{PB}-PH_{2}O\right)-P{\mathrm{aCO}}_{2}/\left(R-\left(1-\frac{P{\mathrm{ACO}}_{2}}{P{\mathrm{aCO}}_{2}}\right)\right)-\mathrm{FI}O_{2}∙P{\mathrm{aCO}}_{2}∙\left(1-\frac{1}{R-\left(1-\frac{P{\mathrm{ACO}}_{2}}{P{\mathrm{aCO}}_{2}}\right)}\right) \end{array}$$

The agreement between the modal Pc'O2n identified from the distributions from the model and the modal *ideal* $P{\mathrm{AO}}_{2}$ predicted from Equation 2a was examined across the range of theoretical scenarios modeled and is plotted in Figure 2b. The agreement (relative error and standard deviation) is summarized in Table 3. Overall relative median error was less than 1%, with 95% of measurements lying within 17.5% of the target value.

**TABLE 3:** Systematic modeling: Agreement (variance *r* ^2^, relative median error (%) and 95th centile) between Pc'O2n at the $\dot{V}A/\dot{Q}$ ratio of modal $\dot{V}O_{2}n$ identified from distributions of $\dot{V}O_{2}$ calculated by the multicompartment lung model (modal Pc'O2n), and the predicted modal *ideal* $P{\mathrm{AO}}_{2}$ from Equation 2a, across all scenarios modeled where the resulting $P{\mathrm{aO}}_{2}$ was 50 mm Hg or greater.

$\mathrm{FI}O_{2}$	*r* ^2^	Median error %	95th centile error %
0.18	0.967	1.5	6.3
0.21	0.962	2.1	8.8
0.30	0.955	2.0	14.9
0.50	0.961	1.2	17.4
0.75	0.979	0.5	5.7
1.0	0.995	0.3	1.2
Overall	0.999	0.8	9.1

*Note*: $\mathrm{FI}O_{2}$ was varied from 0.18 to 1.0. At each $\mathrm{FI}O_{2}$, the log standard deviation (log SD) of $\dot{V}A$ was varied in seven increments of 0.25 up to 1.75, repeated for scenarios with an overall $\dot{V}A/\dot{Q}$ ratio of 0.4, 0.8 and 1.6, R = 0.8 and 1.0, and true shunt fraction of 0%, 10% and 20%. See Figure 2b.

B.(ii) Individual patient modeling:

Forty‐one complete sets of measurements were obtained. Data from one patient in the second cohort was excluded from the analysis as there was evidence of inadvertent dilution of the mixed venous blood sample, making calculation of $\dot{V}O_{2}$ unreliable in that patient. $\mathrm{FI}O_{2}$ ranged from 0.35 to 0.80, with a mean (SD) of 0.53 (0.11). $\dot{V}O_{2}$ and $\dot{V}{\mathrm{CO}}_{2}$ were 0.190 (0.061) l/min and 0.178 (0.048) L/min, respectively. Alveolar dead space fraction $\dot{V}\mathrm{DA}/\dot{V}A$ using the Bohr–Enghoff equation was 24.4 (7.6)% and venous admixture $\dot{Q}s/\dot{Q}t$ was 12.4 (6.8)%. Table 4 shows the calculated alveolar O_2_ partial pressures in the patients.

**TABLE 4:** In vivo patient data: Measured end‐tidal O_2_ partial pressure (PE'O2) and calculated and modeled variables in patients in the study (*n* = 41 measurements).

Measured PE'O2 mm Hg	341.1 (79.6)	*p* = 0.6566	
$P{\mathrm{AO}}_{2}$ (from alveolar gas equation using PE'CO2, equation 1) mm Hg	343.3 (82.5)		*p* < 0.0001
$P{\mathrm{AO}}_{2}$ (from alveolar gas equation using $P{\mathrm{aCO}}_{2}$, equation (1a) mm Hg	332.9 (82.1)	*p* < 0.0001	
Modal Pc'O2n (identified from distributions of $\dot{V}O_{2}n$ in model) mm Hg	323.5 (84.6)		*p* = 0.4887 (see Figure 3)
Modal *ideal* $P{\mathrm{AO}}_{2}$ (from equation 2a) mm Hg	321.3 (82.7)		

*Note*: Data are mean values and standard deviation (SD). Statistical significance of comparisons for paired data is shown. $P{\mathrm{AO}}_{2}$, Alveolar O_2_ partial pressure; Modal Pc'O2n, Alveolar‐capillary O_2_ partial pressure at the point of modal or maximal O_2_ uptake rate (modal $\dot{V}O_{2}n$).

**FIGURE 3:**
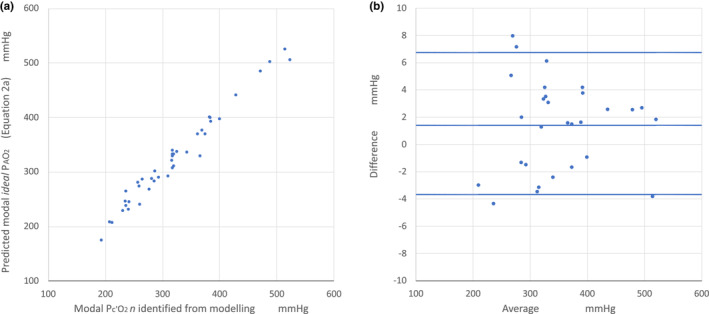
Clinical in vivo individual patient modeling: The relationship of the Pc'O2n identified by modeling in the lung compartment at the modal $\dot{V}O_{2}n$ (modal Pc'O2) to modal *ideal* $P{\mathrm{AO}}_{2}$ predicted by Equation 2a in the patients studied (*n* = 41 measurements) as a correlation plot (Figure 3a). $\mathrm{FI}O_{2}$ ranged from 0.35 to 0.80. The corresponding Bland–Altman plot is shown in Figure 3b.

Primary endpoint: The predicted modal *ideal* $P{\mathrm{AO}}_{2}$ calculated from Equation 2a was similar in magnitude to the modal Pc'O2n identified from the model (mean (SD) 321.3 (82.7) versus 323.5 (84.6), *p* = 0.4887). The comparison for all patients is shown in Figure 3a,b. *r* ^2^ was 0.973, and the median (95th centile) relative error was 1.5 (9.0)%. Mean bias was 1.4 mm Hg with upper and lower 95% limits of agreement of 6.7 and − 3.7 mm Hg, respectively. This confirmed in vivo the agreement demonstrated by the theoretical modeling data shown in Figure 2b and Table 3.

Secondary Endpoints: Mean $P{\mathrm{AO}}_{2}$ from the alveolar gas equation (Equation 1) using measured PE'CO2 as $P{\mathrm{ACO}}_{2}$ was not different to the measured PE'O2 (mean (SD) 341.1 (79.6) versus 343.3 (82.5), *p* = 0.6566), which confirmed the basis for the derivation of alveolar gas equation given in Appendix 1. Substitution of $P{\mathrm{ACO}}_{2}$ with measured $P{\mathrm{aCO}}_{2}$ (Equation 1a) resulted in a calculated mean $P{\mathrm{AO}}_{2}$ which was 10.4 mm Hg lower. Both these variables were significantly higher than the mean modal Pc'O2n identified from the multicompartment model (323.5 mm Hg) for each patient (*p* < 0.0001).

## 4 DISCUSSION

This study describes and validates a mathematical definition of ideal alveolar oxygen partial pressure based on consideration of realistic, physiological distributions of ventilation, blood flow, and respiratory gas exchange in the lung. As shown by modeling of distributions using both theoretical and in vivo data, it can be calculated with adequate precision from a relatively simple modification of the alveolar gas equation which accounts for wide variation in $\dot{V}A/\dot{Q}$ throughout the lung, and in $\mathrm{FI}O_{2}$, using readily measured clinical variables ($\mathrm{FI}O_{2}$, and end‐tidal and arterial CO_2_ partial pressures). This adds to recent work providing a modal definition of ideal alveolar gas for CO_2_ and inert gases, and further reconciles the concept of an ideal alveolar partial pressure with modern understanding of physiological distributions of ventilation, blood flow, and gas exchange in the lung.

The O_2_ partial pressure at the $\dot{V}A/\dot{Q}$ ratio where the distribution of $\dot{V}O_{2}$ in a lung is maximal (modal) is a rational definition of “ideal” alveolar O_2_ within any lung with a given degree of $\dot{V}A/\dot{Q}$ scatter. On either side of this point, increasing or decreasing $\dot{V}A/\dot{Q}$ ratios result in falling effectiveness of O_2_ uptake. In this sense, the concept shares the same principle as the central compartment of the three‐compartment model of $\dot{V}A/\dot{Q}$ heterogeneity (Peyton, 2021). However, by contrast, it does not propose a single, uniform compartment where all lung gas exchange is assumed to take place. Instead, it identifies an “ideal” point on a more physiologically realistic continuum of $\dot{V}A/\dot{Q}$ ratios and gas exchange in the lung.

The traditional theoretical “ideal” compartment of the three‐compartment model is defined as containing all $\dot{V}O_{2}$ and $\dot{V}{\mathrm{CO}}_{2}$. However, Figure 1, which plots realistic, if idealized, distributions of $\dot{V}A$, $\dot{Q}$ and resulting gas exchange, throws this concept into doubt. Due to the very different solubilities of O_2_ and CO_2_ in blood, $\dot{V}O_{2}$ and $\dot{V}{\mathrm{CO}}_{2}$ distributions are not colocated in the lung. Figure 1 shows that when $\dot{V}A/\dot{Q}$ scatter becomes substantial, distributions of $\dot{V}O_{2}$ and $\dot{V}{\mathrm{CO}}_{2}$ diverge across a wider range of real $\dot{V}A/\dot{Q}$ ratios. The assumption that O_2_ and CO_2_ share a common “ideal” compartment, and the same alveolar dead space fraction, is thus not supported by modeling of physiological distributions. This has been shown to be the case in anesthetized patients, where measured alveolar dead space, and conversely “effective” or ideal alveolar volume, for CO_2_ and a range of inert anesthetic gases varied widely, in inverse relationship to their solubility in blood (Peyton et al., 2020). Similar contradictions are encountered in consideration of ideal alveolar gas for shunt fraction calculation with increasing $\dot{V}A/\dot{Q}$ scatter using the three‐compartment model.

By contrast, the mass balance calculation employed in the Riley model to derive Equation 1 is given in Appendix 1C. The “ideal” compartment envisaged in the Riley model, with ventilation $\dot{V}A\text{ideal}$, is considered to contain all $\dot{V}O_{2}$ and $\dot{V}{\mathrm{CO}}_{2}$. However, Figure 1 shows that $\dot{V}O_{2}$ occurs predominantly across a lower range of values of $\dot{V}A/\dot{Q}$ ratios than $\dot{V}{\mathrm{CO}}_{2}$. Thus, any “ideal” compartment which captures all $\dot{V}{\mathrm{CO}}_{2}$ must contain higher $\dot{V}A/\dot{Q}$ ratios that would form part of alveolar dead space for O_2_. This means that $P{\mathrm{AO}}_{2}\text{ideal}$ calculated in Equation 1 must be contaminated with alveolar dead space gas for O_2_ (with partial pressure PIO_2_).

In fact Figure 1 shows how, in a lung with significant $\dot{V}A/\dot{Q}$ scatter such as studied here, the volume of the alveolar compartment that captures all $\dot{V}O_{2}$ and $\dot{V}{\mathrm{CO}}_{2}$ most closely approximates that of total alveolar ventilation $\dot{V}A$. The data in Table 4 from the patient sample studied here, showing the equivalence of PE'O2 with $P{\mathrm{AO}}_{2}\text{ideal}$ calculated from Equation 1, is consistent with this. The derivation in Appendix 1A appropriately reflects this and calculates mixed alveolar gas partial pressure, which includes the entire content of the alveolar dead space compartment as well as the ideal compartment. This manifests as end‐tidal partial pressure in healthy lungs with no longitudinal gas flow stratification and a flat end‐expiratory Phase 3 gas concentration plateau. Note that in the absence of $\dot{V}A/\dot{Q}$ scatter, where there are no significant alveolar‐arterial partial pressure gradients for CO_2_, Equation 2a will indeed simply approximate Equation 1a.

Thus, the Riley model does not provide a satisfactory basis for identifying or measuring the content of a central “ideal” compartment, even though this is fundamental to calculation of venous admixture and dead space using the shunt and Bohr equations, (Berggren, 1942; Bohr, 1891) and the alveolar gas equation is still commonly referred to as the “ideal alveolar gas” equation (West et al., 2020). In their seminal manuscript of 1949, Riley and Cournand presented a “Concept of ‘ideal’ alveolar air” using a representation of the three‐compartment lung model (Riley & Cournand, 1949). This incorporated a pulmonary shunt and an “ideal” uniform gas exchanging compartment, but considered only a single dead space compartment that included serial (anatomic) dead space. Indeed, the authors pointed out that their derivation of the mass balance alveolar gas equation arising from this model was predicated upon homogenous lung gas exchange. Their model effectively ignored the possibility of a separate alveolar dead space compartment arising from $\dot{V}A/\dot{Q}$ heterogeneity. This can be identified by distinguishing the content of end‐tidal gas from mixed expired gas, something that was technically difficult to do in that day, but is now readily done during routine clinical monitoring of tidal gas concentrations using rapid response gas analyzers.

The common substitution of PE'CO2 with $P{\mathrm{aCO}}_{2}$ in the alveolar gas equation (Equation 1a) only increased this confusion by further distancing the use of the alveolar gas equation from its original derivation. Interestingly, however, the use of $P{\mathrm{aCO}}_{2}$ in the numerator of Equation 2a makes sense when employing the modal definition, as it more closely approximates the Pc'CO2n at the modal $\dot{V}O_{2}n$ point as can be seen in Figure 1.

Riley's approach was further constrained by the implicit assumption made by all authors in the field that alveolar O_2_ and CO_2_ partial pressures shared not just a common central “ideal” compartment, but a common ideal point on the “O_2_‐CO_2_” or Fenn diagram, which describes the range of all possible alveolar partial pressures for O_2_ and CO_2_ that can be present for any given combination of inspired and mixed venous partial pressures, at all possible $\dot{V}A/\dot{Q}$ ratios between zero and infinity. In contrast to the three‐compartment model, the Fenn diagram models a continuum of possible gas exchange states across the lung (Fenn et al., 1946). The assumption of a common “ideal point” for the two respiratory gases has heavily influenced thinking on gas exchange theory. Riley's theory proposed that this ideal $\dot{V}A/\dot{Q}$ ratio was located where R*n* is equal to overall lung R. This point is indicated in Figure 1 and lies between the modal $\dot{V}O_{2}n$ and $\dot{V}{\mathrm{CO}}_{2}n$ points. There is no feature to suggest a common “ideal” characteristic of the partial pressures for both gases at this $\dot{V}A/\dot{Q}$ ratio, as has been recently demonstrated by other authors using a simpler 2‐compartment model of $\dot{V}A/\dot{Q}$ mismatch (Wagner et al., 2022).

By contrast, the modal *ideal* definition accepts different distributions of gas exchange in the lung for gases of differing solubility, and different ideal points. The positions of these gas exchange distributions, skewed toward lower $\dot{V}A/\dot{Q}$ ratios for less soluble gases such as O_2_, represent different degrees of “wasted ventilation” and therefore of alveolar dead space. This results in vastly different alveolar dead space fractions for gases of differing solubility, which has been shown when comparing CO_2_ and soluble anesthetic gases in anesthetized patients (Peyton et al., 2020). This implies very different sized ideal compartments and effective alveolar ventilation rates for different gases simultaneously within any given patient, which is not consistent with the concept of a common ideal point or lung compartment for different gases along the $\dot{V}A/\dot{Q}$ axis.

The modal definition of the ideal partial pressure was recently described for CO_2_, and a simple modification of the Bohr equation for calculation of dead space fraction was validated in the same population as the current study (Peyton, 2021). That investigation showed that the modal *ideal* $P{\mathrm{ACO}}_{2}$ was equal to the mean of the arterial and mixed alveolar or end‐tidal CO_2_ partial pressures, across a wide range of $\dot{V}A/\dot{Q}$ scatter and overall $\dot{V}A/\dot{Q}$ ratios. This was also the case for all inert gases, which have linear blood gas dissociation curves. Thus, for CO_2_

(4) $$\begin{array}{c} \text{modal}\;\text{ideal}\;P{\mathrm{ACO}}_{2}=\left(P{\mathrm{aCO}}_{2}+\mathrm{PE}\;'{\mathrm{CO}}_{2}\right)/2 \end{array}$$

The accuracy of Equation 2a in predicting modal *ideal* $P{\mathrm{AO}}_{2}$ is relatively robust in presence of varying degrees of true shunt. Redistribution of a proportion of total pulmonary blood flow to a true shunt compartment had little a priori effect on the position of the modal Pc'O2n point in the modeling. However, Equations 3 and 2a use arterial‐alveolar CO_2_ partial pressure gradients, which are relatively unaffected by true shunt compared to O_2_ partial pressures, to estimate R*n* from R. However, a limitation of the study is that the accuracy of Equation 3 in predicting modal R*n* at the modal $\dot{V}O_{2}n$ point only remained acceptable in the theoretical lung modeling where the resulting $P{\mathrm{aO}}_{2}$ of the scenario was 50 mm Hg or more. In scenarios simulating more profound degrees of hypoxemia, this relationship ceased to provide a satisfactory prediction of R*n*, due to the flattening of the slope of the O_2_ dissociation curve relative to that for CO_2_ in an increasing proportion of lower $\dot{V}A/\dot{Q}$ lung units with very low Pc'O2n values. This may limit its application in altitude physiology, for instance. It should be noted that the linearity of estimation of alveolar PO_2_ by the traditional alveolar gas equation in the presence of severe hypoxemia has not been systematically assessed by modeling in the past, although the variability in calculation of shunt fraction with variation in physiological factors such as PIO_2_ or cardiac output has been the subject of a number of studies (Lynch et al., 1979; Quan et al., 1980; Whiteley et al., 2002).

A further limitation of this study is that the clinical validation of the theory presented is conducted in an anesthetized population, where $\mathrm{FI}O_{2}$ was maintained above 0.35 in accordance with standard safe intraoperative management. Thus, the results presented of modeling at lower $\mathrm{FI}O_{2}$ (0.18 and 0.21) are not directly confirmed. Nevertheless, when Equation 2a is used for estimation of blood O_2_ content in the ideal compartment, it is expected to lead to a smaller calculated shunt fraction (for example, approximately 15% smaller in relative terms for the scenario depicted in Figure 1) than a result based on the customary alveolar gas equation, which will overestimate ideal $P{\mathrm{AO}}_{2}$ in comparison due to its inability to distinguish the contributions of “ideal” and alveolar dead space lung compartments. This difference in shunt calculation will be relatively larger again at lower $\mathrm{PI}O_{2}$, such as room air breathing or altitude, than in the population studied here.

Despite this limitation, the data in Table 4 illustrate the value of studying an anesthetized, ventilated population with normal underlying lung function, when exploring the effects of $\dot{V}A/\dot{Q}$ mismatch on alveolar‐capillary gas exchange. This population has substantial degrees of $\dot{V}A/\dot{Q}$ scatter, but with relatively flat end‐expired gas concentration curves (see Figure S1). Using modern rapid gas analyzers, mixed alveolar gas partial pressures can therefore be estimated with reasonable precision from end‐tidal gas sampling. This technology and study population were not available when many of the seminal works of respiratory physiology of the postwar era were being published, nor was the computing technology that allows distributions such those in Figure 1 to be generated and studied. Riley and Cournand lamented that it was unfortunate at the time that “mixed alveolar air and mixed capillary blood cannot be determined with accuracy” (Riley & Cournand, 1949). Subsequently, reliance on study of healthy awake subjects with little $\dot{V}A/\dot{Q}$ heterogeneity on the one hand, and of patients with lung disease with disturbed expiratory gas flow on the other, has obscured the anomalies underlying the use of the alveolar gas equation to estimate “ideal” alveolar gas.

With this in mind, it should be noted that the modal definition for *ideal* alveolar gas is based upon consideration of alveolar‐capillary gas exchange distribution in the presence of $\dot{V}A/\dot{Q}$ heterogeneity. It should not be confused with other factors, such as longtitudinal or “stratified” ventilatory heterogeneity commonly seen in lung disease or airflow obstruction, which distort the expirogram for CO_2_ and other gases. These factors, largely concerned with gas flow limitation and heterogeneity in the conducting airways, may prompt controversies about the most appropriate point on the expiratory gas concentration curve to define alveolar partial pressure, for example (Tusman et al., 2012). However, such discussions should be seen as separate to the theory of alveolar‐capillary gas exchange on which the modal *ideal* point is based.

In conclusion, an alternative “modal” definition of ideal alveolar oxygen partial pressure is described, based on realistic physiological distributions of alveolar ventilation and lung blood flow, at the $\dot{V}A/\dot{Q}$ ratio where the distribution of oxygen uptake rate is maximal or modal. The modal ideal alveolar oxygen partial pressure is able to be determined with accuracy across a wide range of $\dot{V}A/\dot{Q}$ heterogeneity and $\mathrm{FI}O_{2}$, using a simple modification of the alveolar gas equation (Equation 2a) and should be considered as a more coherent solution to the problem of calculation of ideal alveolar oxygen.

## AUTHOR CONTRIBUTIONS

Philip Peyton conceived and designed research; performed experiments; analyzed data; interpreted results of experiments; prepared figures; drafted manuscript; edited and revised manuscript; and approved final version of manuscript.

## FUNDING INFORMATION

This work was supported by a Project Grant DJ17/006 from the Australian and New Zealand College of Anaesthetists Research Foundation.

## CONFLICT OF INTEREST STATEMENT

Philip Peyton has received research consultancy payments from Maquet Critical Care/Getinge for an unrelated project.

## Supporting information


Figure S1

## Data Availability

Expressions of interest for data availability can be directed to the author.

## APPENDIX 1

### A: Derivation of the alveolar gas equation for the whole lung

The derivation of the alveolar gas equation is based upon simple mass balance in the lung, and here uses the expanded form of the alveolar gas equation which incorporates adjustment for the differences between inspired and expired alveolar tidal ventilation volumes. Barometric pressure PB is considered to be corrected for saturated water vapor pressure.

In a lung ventilated with a given $\mathrm{FI}O_{2}$, oxygen uptake rate $\dot{V}O_{2}$ is given by

$$\dot{V}\mathrm{AI}∙{\mathrm{FIO}}_{2}∙\mathrm{PB}-\dot{V}A∙P{\mathrm{AO}}_{2}=\dot{V}O_{2}∙\mathrm{PB}$$

and CO_2_ elimination rate $\dot{V}{\mathrm{CO}}_{2}$ is given by

$$\dot{V}A∙P{\mathrm{ACO}}_{2}=\dot{V}{\mathrm{CO}}_{2}∙\mathrm{PB}$$

where $P{\mathrm{AO}}_{2}$ and $P{\mathrm{ACO}}_{2}$ are mixed alveolar partial pressures of O_2_ and CO_2_, $\dot{V}A$ is expired alveolar ventilation rate and inspired alveolar ventilation rate $\dot{V}\mathrm{AI}$ is

$$\dot{V}\mathrm{AI}=\dot{V}A+\dot{V}O_{2}–\dot{V}{\mathrm{CO}}_{2}$$

Given that the respiratory exchange ratio R is

$$R=\dot{\dot{V}{\mathrm{CO}}_{2}/}\dot{V}O_{2}$$

then

$$P{\mathrm{AO}}_{2}={\mathrm{FIO}}_{2}∙\mathrm{PB}-P{\mathrm{ACO}}_{2}/\left(R-\left[{\mathrm{FIO}}_{2}∙P{\mathrm{ACO}}_{2}\right)∙\left(1-\frac{1}{R}\right)\right]$$

The term in square brackets is often left out to provide the simplified form of the alveolar gas equation (where the difference between $\dot{V}\mathrm{AI}$ and $\dot{V}A$ in the lung overall is ignored).

Note that substitution of $P{\mathrm{ACO}}_{2}$ with $P{\mathrm{aCO}}_{2}$ is commonly done in practice:

$$P{\mathrm{AO}}_{2}={\mathrm{FIO}}_{2}∙\mathrm{PB}-P{\mathrm{aCO}}_{2}/\left(R-\left[{\mathrm{FIO}}_{2}∙P{\mathrm{aCO}}_{2}\right)∙\left(1-\frac{1}{R}\right)\right]$$

### B: Calculation in any lung compartment

Within any lung compartment *n*, the same mass balance derivation applies.

$$\begin{array}{c} \dot{V}\mathrm{AI}n\;∙\;{\mathrm{FIO}}_{2}∙\mathrm{PB}-\dot{V}An\;∙\;\mathrm{Pc}\;'O_{2}n=\dot{V}O_{2}n\;∙\;\mathrm{PB} \end{array}$$

and CO_2_ elimination $\dot{V}{\mathrm{CO}}_{2}n$ is given by

$$\dot{V}An∙\mathrm{Pc}\;'{\mathrm{CO}}_{2}n=\dot{V}{\mathrm{CO}}_{2}n∙\mathrm{PB}$$

where Pc'O2n and Pc'CO2n are equilibration alveolar‐capillary partial pressures of O_2_ and CO_2_, $\dot{V}An$ is expired alveolar ventilation rate in lung compartment *n*, and inspired alveolar ventilation rate $\dot{V}\mathrm{AI}n$ is

$$\dot{V}\mathrm{AI}n=\dot{V}An+\dot{V}O_{2}n–\dot{V}{\mathrm{CO}}_{2}n$$

Given that the respiratory exchange ratio R*n* for lung compartment *n* is

$$Rn=\dot{V}{\mathrm{CO}}_{2}n/\dot{V}O_{2}n$$

then

(A1*n*)
$$\begin{array}{c} \mathrm{Pc}\;'O_{2}n=\mathrm{FI}O_{2}∙\mathrm{PB}-\mathrm{Pc}\;'{\mathrm{CO}}_{2}n/Rn-\left[\mathrm{FI}O_{2}∙\mathrm{Pc}\;'{\mathrm{CO}}_{2}n∙\left(1-1/Rn\right)\right] \end{array}$$

In similar fashion to Equation A1, approximation of Pc'CO2n with Pc'CO2 gives

$$\begin{array}{c} \mathrm{Pc}\;'O_{2}n=\mathrm{FI}O_{2}∙\mathrm{PB}-P{\mathrm{aCO}}_{2}n/Rn-\left[\mathrm{FI}O_{2}∙P{\mathrm{aCO}}_{2}n∙\left(1-1/Rn\right)\right] \end{array}$$

### C: Three‐compartment (Riley) model calculation

Within an “ideal” lung compartment, the same mass balance derivation applies where all respiratory gas exchange ($\dot{V}O_{2}$ and $\dot{V}{\mathrm{CO}}_{2}$) is considered to take place.

$$\begin{array}{c} \dot{V}\text{AI}\text{ideal}∙{\mathrm{FIO}}_{2}∙\mathrm{PB}-\dot{V}A\text{ideal}∙P{\mathrm{AO}}_{2}\text{ideal}=\dot{V}O_{2}∙\mathrm{PB} \end{array}$$

and CO_2_ elimination $\dot{V}{\mathrm{CO}}_{2}$ is given by

$$\dot{V}A\text{ideal}∙P{\mathrm{ACO}}_{2}\text{ideal}=\dot{V}{\mathrm{CO}}_{2}∙\mathrm{PB}$$

Given that

$$\dot{V}\mathrm{AI}\text{ideal}=\dot{V}A\text{ideal}+\dot{V}O_{2}–\dot{V}{\mathrm{CO}}_{2}$$

and

$$R=\dot{V}{\mathrm{CO}}_{2}/\dot{V}O_{2}$$

then

(A1)
$$P{\mathrm{AO}}_{2}\text{ideal}=\mathrm{FI}O_{2}∙\mathrm{PB}-P{\mathrm{ACO}}_{2}\text{ideal}/R-\left[\mathrm{FI}O_{2}∙P{\mathrm{ACO}}_{2}\text{ideal}∙\left(1-1/R\right)\right]$$

In similar fashion to above, approximation of $P{\mathrm{ACO}}_{2}\text{ideal}$ with $P{\mathrm{aCO}}_{2}$ gives

(A1a)
$$P{\mathrm{AO}}_{2}\text{ideal}=\mathrm{FI}O_{2}∙\mathrm{PB}-P{\mathrm{aCO}}_{2}/R-\left[\mathrm{FI}O_{2}∙P{\mathrm{aCO}}_{2}∙\left(1-1/R\right)\right]$$

## APPENDIX 2

### Multicompartment model structure

Model of theoretical distributions of $\dot{V}A$ and $\dot{Q}$: The multicompartment computer model of $\dot{V}A/\dot{Q}$ scatter used in the study generated unimodal idealized lognormal distributions across N lung compartments *n* of blood flow $\left(Qn\right)$ and expired alveolar ventilation $\dot{V}A$ *n*, with the degree of $\dot{V}A/\dot{Q}$ scatter quantified by the log standard deviation (log SD) of the distribution of either $\dot{V}A$ and $\dot{Q}$ according to the method described by West (Kelman, 1966, 1967; Olszowska & Wagner, 1980; Peyton et al., 2020). Hundred lung compartments were used for the current study to minimize quantization error in output variables from the model. A proportion of blood flow could be diverted to an additional true shunt compartment. The model makes no attempt to incorporate longtitudinal inhomogeneity or stratification of ventilation distribution, and focuses solely on alveolar‐capillary gas exchange calculations.

For any given set of distributions of $\dot{V}A$ and $\dot{Q}$, steady‐state gas exchange assuming a continuous flow principle with full equilibration between blood and alveolar gas in each lung compartment, was computed from mass balance according to the Fick principle simultaneously for every alveolar gas using an iterative method, as described by Olszowska and Wagner (Siggaard‐Andersen, 1974), incorporating the dissociation curve for O_2_ and CO_2_ according to the equations of Kelman (Fenn et al., 1946; Wagner et al., 2022) using a previously published algorithm. This determines interdependent oxygen and carbon dioxide partial pressures across a wide range of $\dot{V}A/\dot{Q}$ ratios and compartmental acid‐base status using arterial base excess (BE) incorporating the formula described by Siggaard‐Andersen (Kelman, 1966; Quan et al., 1980).

Inputs to the calculation were inspired and mixed venous partial pressures for each gas *G* (PI*G* and Pv*G*). For each gas species, O_2_, CO_2_, and nitrogen, gas exchange in each lung compartment *n* ($\dot{V}Gn$) was calculated according to the mass balance principle

$$\begin{array}{c} \dot{V}\mathrm{Gn}∙\mathrm{PB}=\dot{V}\mathrm{AI}n∙\mathrm{PI}G–\dot{V}An∙{\mathrm{Pc}}^{\prime }\mathrm{Gn}=\dot{Q}∙\left({\mathrm{Cc}}^{\prime }\mathrm{Gn}–C\bar{v}G\right) \end{array}$$

Where $\dot{V}\mathrm{AI}n$ was the inspired alveolar ventilation rate for compartment *n*, Pcʹ*Gn*, and Ccʹ*Gn* were equilibrated partial pressures of alveolar gas and end‐capillary blood and corresponding end‐capillary blood gas content, respectively, $C\bar{v}G$ was mixed venous blood content of *G*, and where for each inert gas, Ccʹ*Gn* = λb/g*G* Pcʹ*Gn*/PB and $C\bar{v}G=\mathrm{\lambda b}/\mathrm{gG}∙P\bar{v}G/\mathrm{PB}$.

Outputs from the model were the distributions of Pcʹ*Gn*, Ccʹ*Gn*, and $\dot{V}Gn$ of each gas. Mixed alveolar gas partial pressure (PA*G*) and arterial partial pressure (Pa*G*) and content (Ca*G*) were calculated from the flow weighted means of these outputs from all lung compartments, including mixed venous blood from true shunt, according to

$$\Sigma \left(\dot{V}An∙{\mathrm{Pc}}^{\prime }\mathrm{Gn}\right)/\Sigma \dot{V}An=\mathrm{PA}G$$

$$\Sigma \left(\dot{Q}n∙{\mathrm{Cc}}^{\prime }\mathrm{Gn}\right)/\Sigma \dot{Q}n=\mathrm{Ca}G$$

and total gas exchange for each gas ($\dot{V}G$) was

$\dot{V}G=\Sigma \dot{V}\mathrm{Gn}$.

Because measurements for total oxygen uptake rate ($\dot{V}O_{2}$) and CO_2_ elimination rate ($\dot{V}{\mathrm{CO}}_{2}$) were made in all patients, a further step was added where total inspired $\dot{V}\mathrm{AI}$, mixed venous partial pressures $P\bar{v}O_{2}$, and $P\bar{v}CO_{2}$ were varied iteratively using modified continuous bisection so as to achieve the target values. The model was constructed on a graphical user interface using LabVIEW 2011 (National Instruments, TX) (Kelman, 1966; Olszowska & Wagner, 1980).

Shunt fraction ($\dot{Q}s/\dot{Q}t$) was calculated from O_2_ content in blood using the shunt equation of Berggren.

$$\dot{Q}s/\dot{Q}t=\frac{{\mathrm{Cc}}^{\prime }O_{2}–\mathrm{Ca}O_{2}}{{\mathrm{Cc}}^{\prime }O_{2}–C\bar{v}O_{2}}$$

End‐capillary oxygen content ${\mathrm{Cc}}^{\prime }O_{2}$ was obtained from the equations of Kelman which estimate O_2_ hemoglobin saturation ScʹO_2_ from oxygen partial pressure, using ideal $P{\mathrm{AO}}_{2}$ from Equation A1.

$$\begin{array}{c} {\mathrm{Cc}}^{\prime }O_{2}=1.34∙{\mathrm{Sc}}^{\prime }O_{2}+0.003∙P{\mathrm{AO}}_{2} \end{array}$$

Alveolar dead space fraction was calculated using the Enghoff modification of the Bohr equation.

$$\mathrm{VDA}/\mathrm{VA}=1-P{\mathrm{ACO}}_{2}/P{\mathrm{aCO}}_{2}$$

## APPENDIX 3

### Clinical in vivo data collection protocol

Anesthesia management consisted of routine patient cardiovascular monitoring with placement of peripheral arterial and pulmonary artery catheters, along with continuous tidal gas concentration monitoring and arterial and mixed venous blood gas analysis. Following intravenous induction of anesthesia and tracheal intubation, the patient was connected to a circle breathing system with fresh carbon dioxide absorber to eliminate carbon dioxide rebreathing. Tidal volume was set at 7–10 mL/kg, and respiratory rate set so as to achieve an end‐tidal carbon dioxide partial pressure ($P{\text{EtCO}}_{2}$) of 25–35 mm Hg, with an inspired to expired (I:E) ratio that ensured a smooth and flat phase 3 plateau on the capnogram, allowing accurate determination of end‐tidal gas partial pressures. Maintenance anesthesia consisted of inhalational anesthetic agent without nitrous oxide. Delivered concentrations of oxygen were set to achieve an $\mathrm{FI}O_{2}$ of 0.30–0.50 in the first study and 0.50–0.60 in the second study, with anesthesia provided by a volatile anesthetic agent with or without supplementary intravenous propofol infusion and opioid to maintain adequate depth of anesthesia.

During near steady‐state maintenance phase anesthesia, between 30 and 90 min postinduction of anesthesia and prior to cardiopulmonary bypass, and during a period of stability (less than 10% change over at least a 5 min period of time) in ventilation settings, heart rate, mean arterial blood pressure, and end‐expired concentration of carbon dioxide and volatile agent, cardiac output ($\dot{Q}t$) was measured by right heart thermodilution, and simultaneous paired arterial and mixed venous blood samples were collected, along with recording of inspired and end‐tidal concentrations of oxygen and carbon dioxide by sidestream gas sampling using a Datex‐Ohmeda Capnomac Ultima gas analyzer (GE Healthcare, Madison WI) calibrated across the full range of measured gas concentrations encountered. End‐tidal concentrations were measured at the midpoint of the Phase 3 plateau of the CO_2_ expirogram, which approximates the point recommended by recent authors as that representing mixed or mean alveolar gas even in the presence of a phase 3 slope. This was connected to a notebook computer (Macbook Air, Apple Corp, Cupertino, CA) running LabVIEW software (National Instruments, Austin TX), via an analog‐digital converter card (USB 6009, National Instruments, Austin TX). Blood samples were processed for measurement of respiratory blood gases on a Radiometer ABL gas analyzer calibrated according to manufacturer's specifications.

$\dot{V}{\mathrm{CO}}_{2}$ and $\dot{V}O_{2}$ were calculated from measured thermodilution cardiac output $\dot{Q}t$ using the Fick equation and from the measured values obtained on blood gas analysis for arterial and mixed venous O_2_ and CO_2_ content. $\dot{V}A$ was calculated from the ratio of $\dot{V}{\mathrm{CO}}_{2}$ to end‐tidal CO_2_ concentration. $P{\mathrm{AO}}_{2}$ was calculated from Equation A1 and Equation A1a. End‐capillary oxygen content ${\mathrm{Cc}}^{\prime }O_{2}$ was obtained from the equations of Kelman which estimate O_2_ hemoglobin saturation from oxygen partial pressure, using the $P{\mathrm{AO}}_{2}$ from Equation A1. (Peyton et al., 2001) Shunt fraction ($\dot{Q}s/\dot{Q}t$) was then calculated using the shunt equation.
